# Corrigendum

**DOI:** 10.1111/pbi.14044

**Published:** 2023-04-11

In the article “Genetic variation in *CaTIFY4b* contributes to drought adaptation in chickpea” by Barmukh *et al*. (2022), the authors wish to add a double affiliation to co‐author Jana Kholova, and insert an additional funding grant in the Acknowledgements section.

The affiliation of Jana Kholova is as follows:

^1^Crops Physiology & Modeling, Accelerated Crop Improvement Research Theme, International Crops Research Institute for the Semi‐Arid Tropics (ICRISAT), Hyderabad, 502324, Telangana, India

^2^Department of Information Technologies, Faculty of Economics and Management, Czech University of Life Sciences Prague, Kamýcká 129, Prague, 165 00, Czech Republic

In the Acknowledgements section, the additional funding grant is:

The Internal grant agency of the Faculty of Economics and Management from the Czech University of Life Sciences Prague, Life Sciences 4.0 Plus, grant no. 2022B0006.
